# Environmental Quality and Aquatic Invertebrate Metrics Relationships at Patagonian Wetlands Subjected to Livestock Grazing Pressures

**DOI:** 10.1371/journal.pone.0137873

**Published:** 2015-10-08

**Authors:** Luis Beltrán Epele, María Laura Miserendino

**Affiliations:** Centro de Investigaciones Esquel de Montaña y Estepa Patagónica (CONICET-UNPSJB), Roca 780 (9200), Esquel, Chubut, Argentina; Chinese Academy of Sciences, CHINA

## Abstract

Livestock grazing can compromise the biotic integrity and health of wetlands, especially in remotes areas like Patagonia, which provide habitat for several endemic terrestrial and aquatic species. Understanding the effects of these land use practices on invertebrate communities can help prevent the deterioration of wetlands and provide insights for restoration. In this contribution, we assessed the responses of 36 metrics based on the structural and functional attributes of invertebrates (130 taxa) at 30 Patagonian wetlands that were subject to different levels of livestock grazing intensity. These levels were categorized as low, medium and high based on eight features (livestock stock densities plus seven wetland measurements). Significant changes in environmental features were detected across the gradient of wetlands, mainly related to pH, conductivity, and nutrient values. Regardless of rainfall gradient, symptoms of eutrophication were remarkable at some highly disturbed sites. Seven invertebrate metrics consistently and accurately responded to livestock grazing on wetlands. All of them were negatively related to increased levels of grazing disturbance, with the number of insect families appearing as the most robust measure. A multivariate approach (RDA) revealed that invertebrate metrics were significantly affected by environmental variables related to water quality: in particular, pH, conductivity, dissolved oxygen, nutrient concentrations, and the richness and coverage of aquatic plants. Our results suggest that the seven aforementioned metrics could be used to assess ecological quality in the arid and semi-arid wetlands of Patagonia, helping to ensure the creation of protected areas and their associated ecological services.

## Introduction

Wetlands play a critical role in maintaining natural cycles that support biodiversity and provide services that contribute to human well-being and poverty alleviation [1,2]. However, in the Patagonian Region of Argentina, wetland ecosystems receive little attention from ecologists. To date, scientific information about the status of ponds and their associated species, especially aquatic invertebrates, is very scarce. Patagonian wetlands (colloquially known as “mallines”) are associated with a variety of ecosystems, from forests in the West to arid and semiarid steppes in the East. These environments harbor a rich native flora [3], terrestrial and aquatic fauna [4,5], and by providing forage and water supply, they support a regional human economy largely based on livestock breeding. Since the first European settlers colonized Patagonia (between 1880 and 1920), extensive livestock grazing has become so widespread that to date, ungrazed areas are practically nonexistent. Additionally, inadequate management of this region has resulted in a preponderance of land with such low productivity that landowners have been forced to switch from extensive to intensive grazing practices in locally productive areas such as wetland pastures [6].

Several authors suggest that even low levels of grazing can alter wetland functions through herbivory of aquatic vegetation, nutrient input via urine and feces deposition, and sedimentation and soil compaction by trampling [7,8]. Decreases in vegetative cover promote the increase of evaporation rates and loss of soils by water and wind erosion [9]. In Patagonia, overgrazing has induced desertification, one of the main environmental problems affecting the region [10]. If climate-change predictions that involve an increase of temperature together with reductions in precipitation are borne out, the result will be a severe reduction of suitable areas for mallines in NW Patagonia [11,12]. In these scenarios, the evaluation of land use effects on wetlands will become extremely significant, particularly in areas that are already classified as arid or semiarid.

According to Balcombe *et al*. [13] given that invertebrates play a vital role in the functioning of wetlands, the comprehensive analysis of these communities can provide an overview of wetland preservation status. Globally, numerous studies in which metrics based on aquatic invertebrate communities have been used to assess wetlands environmental status [13–15]. Aquatic invertebrates are considered valuable as biological indicators because they respond consistently and predictably to anthropogenic disturbances, they are abundant, readily surveyed, and diverse [16]. Moreover, invertebrates are important in wetland food webs [17], so activities that adversely affect them may also affect other trophic levels. In the Patagonia Region, most studies looking for appropriate ecological indicators have been conducted in lotic environments [18], whereas the impacts of anthropogenic activities on invertebrate communities in wetland waters are still relatively unknown.

This study outlines a major approach in the Patagonian wetlands ecological evaluation. Here, comparable data on invertebrate communities was obtained from 30 wetland ponds that were subjected to different levels of grazing intensity. Moreover, we identified the best invertebrate metrics for use in assessing wetlands ecological status. Our main hypothesis is that wetland ponds are strongly influenced by the dominant use of adjacent lands. We also predict that as grazing pressure on wetlands increases, the water quality and the invertebrate communities will be altered. Identifying the consequences of current land use on the mallines of the arid and semiarid areas of Patagonia is a significant challenge. Thus, we consider that this ecological approach to studying mallines would provide significant information to policy-makers, would be useful in the implementation of specific biodiversity conservation actions, and would help to define and maintain the sustainable use of these important ecosystems and the benefits they provide.

## Material and Methods

### Study area

**Fig 1:**
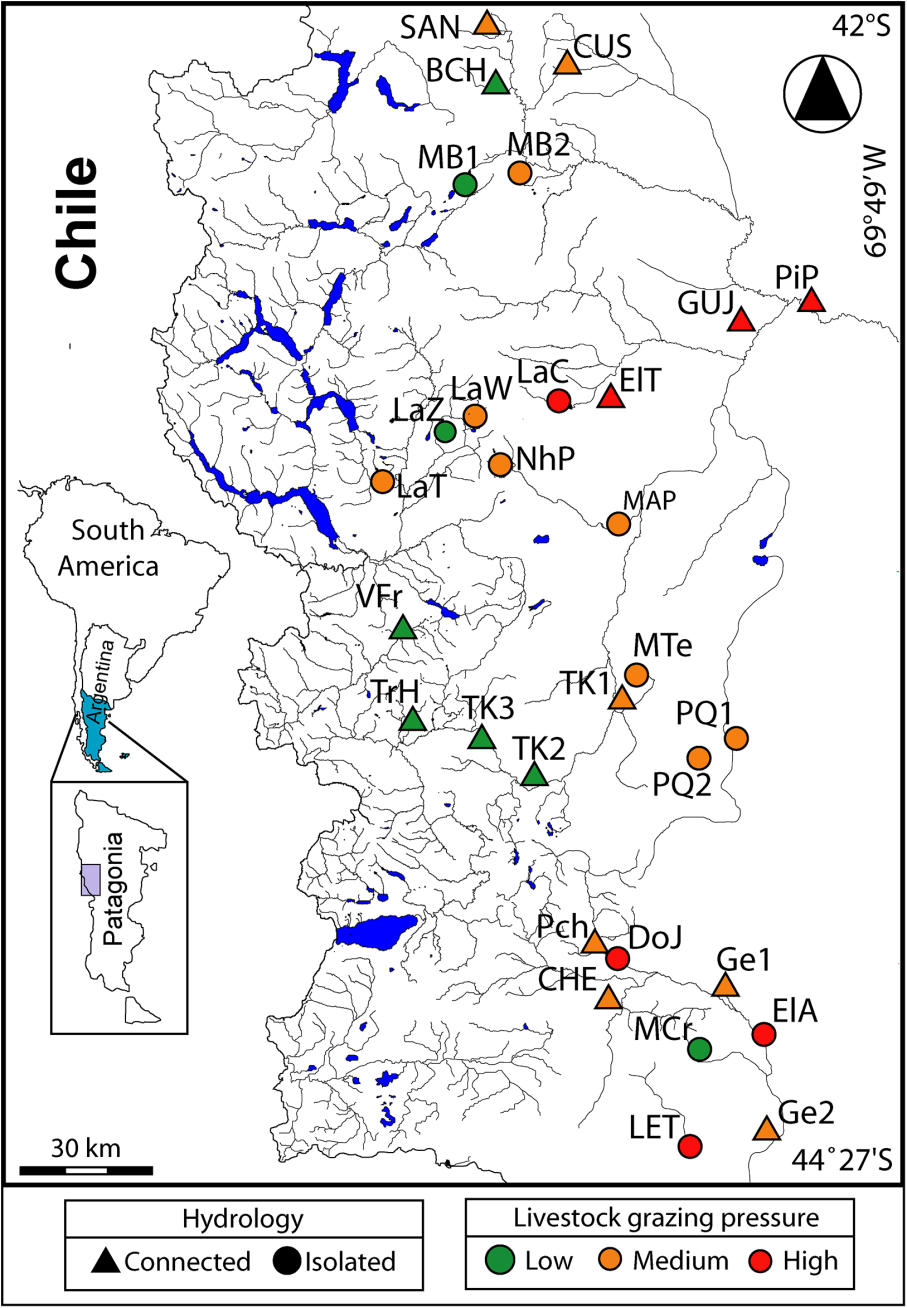
Location of the 30 studied wetlands, Chubut Province, Patagonia, Argentina. Hydrological type and grazing pressure is indicated.

The Argentinean Patagonian region extends from 36° to 55° S. The climate here is generally dry, cold and windy. The study area is located between 42°02’–44°22’ S and 70°13’–71°31’ W (28,600 km^2^) in the transition zone between two phytogeographical provinces (Sub-Antartic Forest and the Patagonian Steppe), which result from the West–East rainfall gradient [19]. Biogeographically, this area is classified as belonging to the Andean–Humid, Sub-Andean Sub-Humid and the Extra-Andean Occidental biozones [10]. All sites are located in NW of Chubut Province, Argentina (Fig 1) where wetlands are very important as water sources and sustain large livestock stocks [8]. Wetlands in these arid and semiarid areas represent 30 to 40% of the forage supply.

### Site selection and land use characterization

A total of 30 wetlands were chosen, based on their accessibility and similarity in land use (pastures), and included small isolated sites, lacunar systems and wetlands associated with rivers and streams (connected). All sites were on private lands a minimum of 5 km from urban areas. Permits to conduct the study on these sites were requested by INTA–Instituto Nacional de Tecnología Agropecuaria. None of the sites had records of endangered or protected species. We attempted to include a wide range of environmental conditions and land use intensities, including representative wetlands from the three different biozones in Chubut Province. Sites were each visited once in two consecutive years (half were sampled in December 2006 and half in December of 2007). Inspection of weather conditions *a posteriori* revealed that both sampling periods were comparable in terms of rain, air temperature, and wind [20]. Surveys were conducted in early summer, the optimal season for obtaining samples representative of local invertebrate diversity and consisting of mature, readily identifiable individuals [21]. Moreover, the natural recharge of pond at wetlands occurs during seasonal rainfalls in winter, and the local ranching practices involve rotating livestock to wetlands in summer for reproductive periods and weaning.

Land use information (livestock raising type and stocking densities) was provided by the governmental agencies Subsecretaría de Bosques y Parques (SubSB) and INTA. All wetlands were used as pasturelands for a mix of livestock types comprised of sheep, cows, and horses. These last, employed in farm labor, also have cultural value for some aboriginal communities [22]. Access of livestock to wetlands was unrestricted at all sites, thus all ponds or water bodies were employed for stock drinking.

The level of grazing pressure at the 30 studied wetlands was assessed as low, medium, or high (Fig 1). Categorization of the level of grazing pressure was based on eight features: 1) livestock density; 2) land carrying capacity score assigned to each wetland following Siffredi *et al*. [23]; 3) evidence of soil erosion and compaction by livestock on the adjacent land; 4) signs of trampling in shorelines and within water bodies; 5) feces amount; 6) signs of foraging on vegetation; 7) presence of exotic plants; and 8) soil salinity. Higher levels of disturbances were assigned to sites with cattle and horses because they physically enter the water bodies. For example, cattle and horses were fed at DoJ wetland year-round; livestock density exceeded the agency INTA recommended; soil was clearly eroded; trampling was evident within and around the shorelines; feces were present in and around ponds; vegetation was heavily foraged. Furthermore, exotic plant species were recorded. As a result, the level of grazing pressure of DoJ was classified as *high*.

### Wetlands environmental features

The hydrologic connectivity of each studied wetland was determined through a set of LandSat 7 images RGB 123 (2003, mapping resolution 30 m × 30 m) and field data. Wetlands were classified as connected or isolated, following Mitsch & Gosselink [24]. Connected wetlands were those located between dry terrestrial systems and permanently flooded deep-water aquatic systems (e.g. rivers, lakes), whereas isolated wetlands were those located in basins with little outflow and no adjacent deep water systems, where groundwater and rainfall were presumed to be the only input. Though we use the term “isolated” it is implicit that they are regularly connected to groundwater [6].

Annual rainfall at each wetland was estimated using the regression models of Jobbágy *et al*. [25] developed for the NW of Chubut Province. The distance to the mountain range was estimated using the Google Earth software.

Sampling effort (environmental features, plant and invertebrates) was mostly concentrated in the inundated zone of wetlands. To evaluate the effect of livestock on abiotic wetland conditions, morphometric and physicochemical measurements were also performed at each site. Measurements included length and width; inundated area (measuring tape or a LandSat 7 images RGB 123); and mean depth (calibrated stick). In shallow ponds, depth was assessed at five points on a line transect, whereas mean depth for lacustrine fringe areas were gathered from the literature [26]. Water temperature (°C), specific conductance (μS/cm), total dissolved solids (mg/L), pH, and dissolved oxygen (mg/L) were obtained with a Hach sensION156 multiparameter probe. To optimize comparisons, site visits were carried out at the same time each day.

At each pond, one water sample (2L) was taken from below the surface and kept at 4°C prior to analysis. In the laboratory, nutrients (±0.01 μg/L) were assessed as follows. Total nitrogen (TN) was assessed via alkaline oxidation with potassium persulphate and boric acid, cadmium column reduction, and subsequent diazotization. Total oxidized nitrogen (NO_3_^–^ plus NO_2_^–^) was obtained following cadmium column reduction and subsequent diazotization. Ammonia (NH_4_^+^) was determined colourmetrically by the indophenol blue method. Total phosphorus (TP) was analyzed by acid oxidation with potassium persulphate and subsequent determination as soluble reactive phosphate (SRP), by the molybdate/ascorbic acid method [27].

### Aquatic plant collection

At each site, a stratified random sampling was performed in an effort to include all aquatic plants life forms present at a site. In permanent environments (deeper sites) the lacustrine fringe areas were examined. Samples were collected and stored in plastic bags. In the laboratory, macrophytes were identified using a regional taxonomic book [28]. A more detailed description of the collection methodology is in Kutschker *et al*. [3]. The percentage of vegetation cover was assessed visually [29] and divided into seven categories (<1%, 1–5%, 6–25%, 26–50%, 51–75%, 75–99%, and 100%).

### Invertebrate analysis

Aquatic invertebrates were sampled using a D-frame net (800 μm mesh). The net was horizontally (1.5m) swept eight times, from the margins to the middle part of the ponds, removing invertebrates associated with epibenthos, nekton, and pleuston. This type of sampling effort attempts to obtain species from most of the habitats within the wetland [16]. Contents of the 8 sweeps were pooled into 1 composite sample. Three composite samples were collected per site. Invertebrates were fixed in the field in 5% formalin. In the laboratory, samples were sorted under 5 x magnification and stored in 70% ethyl alcohol. Organisms were identified using a stereomicroscope (LEICA MZ6) to the lowest possible taxonomic level [30] and counted. By taking into account the area of the D-frame net and the distance covered in the water, we were able to express invertebrate density as the number of individuals per unit volume (ind/m^3^).

For most taxa, available length-mass relationships [31] were used to estimate biomass of individuals. Complete descriptions of methodology and instruments utilized can be found in Epele & Miserendino [32]. Biomass data were expressed as g DM/m^3^. All aquatic invertebrates (adults and larvae) were assigned to functional feeding groups (FFG) using available references [33], knowledge of feeding modes (mouthpart morphology and behaviour), and analysis of gut contents.

### Metric selection

Invertebrate metrics included measures of taxonomic richness, abundance and biomass; tolerance to a given disturbance; percentage taxonomic composition; and functional feeding groups [15,16,34–36]. A total of 36 descriptors based on structural or functional attributes of invertebrates (Table 1) were estimated to assess wetlands quality and to compare sites subjected to different levels of grazing pressure (low, medium or high). However, the total number of metrics employed was 88, as the metrics "insect family biomass" and "invertebrate (no insect) biomass" involved 34 insect families and 15 orders of non-insect invertebrates (S1 Table). Feeding measures were obtained using density and biomass data (a total of 12 metrics).

**Table 1:** Classification and predicted response of wetland invertebrate metrics used in the study.

Measure	Metric	Predicted response to increasing perturbation
**Richness,**	Total taxa	Decrease
**abundance and**	No. of insect families	Decrease
**biomass**	No. of orders of invertebrates no insects	Increase
	No. of aquatic insect taxa	Decrease
	No. of Crustacea taxa	Decrease
	No. of Crustacea + Mollusca taxa	Decrease
	No. of Gastropoda taxa	Decrease
	No. of Hirudinea taxa	Increase
	No. of Coleoptera taxa	Decrease
	No. of Chironomidae taxa	Decrease
	No. of Diptera taxa	Decrease
	Total invertebrate abundance	Increase
	Total invertebrate biomass	Variable
	Insect family biomass	Variable
	Invertebrate (no insect) order biomass	Variable
	Ostracoda abundance	Variable
	Copepoda abundance	Variable
	Cladocera abundance	Variable
**Tolerance**	H’	Decrease
	E	Decrease
**Composition**	% dominant taxon	Increase
	% Hirudinea	Increase
	% Oligochaeta	Increase
	% Crustacea	Increase
	% Amphipoda	Increase
	% EOT	Decrease
	% Ephemeroptera	Decrease
	% Ephemeroptera+Trichoptera	Decrease
	% Dytiscidae	Decrease
	% Diptera	Increase
	% Chironomidae	Increase
	% Orthocladiinae in Chironomidae	Decrease
	% Gastropoda	Decrease
**Feeding groups** *	% Predators	Variable
	% Scrapers	Decrease
	% Filterers	Decrease
	% Collector–gatherers	Variable
	% Shredders	Decrease
	% Piercers–herbivores	Variable

EOT: Ephemeroptera, Odonata and Trichoptera; H’: Shannon and Weaver diversity; E: equitativity.

* Feeding groups were analyzed based on the percentage of density and biomass

Following Barbour *et al*. [34], metrics were tested for sensitivity by comparison of low, medium and high impact sites. The sensitivity of each metric (its ability to discriminate between grazing pressure ranks) was judged according to the degree of interquartile overlap in box-and-whisker plots. Metrics were judged to have one of 4 sensitivity values: a sensitivity of 3 (strong) if no overlap existed in the interquartile range; a sensitivity of 2 (strong) if there was some overlap that did not extend to the medians; a sensitivity of 1 (weak) if there was a moderate overlap of interquartile ranges but at least 1 median was outside the range; and a sensitivity of 0 if the interquartile overlap was considerable; with no discrimination between reference and impaired sites. We chose to omit from our model those metrics that did not show the expected response to disturbance and did not segregate sites with different impact levels or that showed many zeros in the matrices (e.g. occurred when the evaluated metric is based on a species or trait that was uncommon in the assessed sites).

### Statistical analysis

A Pearson correlation matrix was used to reveal any covariation between environmental features (STATISTICA 6.0). When variables displayed a strong covariation (R > 0.4; *p* < 0.05), one was only retained.

Principal component analysis (PCA) was performed in order to identify major sources of variation in physical and chemical variables across the 30 studied wetlands. Prior to analysis, all environmental data (except pH) were log (*x*+1) transformed [37].

After applying the Barbour *et al*. [34] procedure, the set of selected metrics were tested to determine their agreement with the following criteria: 1) to be non-redundant, 2) to show the predicted response to water quality variables, 3) to display the highest sensitivity to grazing pressure, 4) to be user-friendly for future monitoring purposes.

Redundancy among metrics was tested using a Pearson correlation analysis in STATISTICA 6.0. A metric was considered redundant if the correlation coefficient among the metrics set was higher than 0.75, and the *p*-value smaller than 0.05. Relationships among the candidate metrics and physicochemical variables were assessed graphically (Pearson correlation coefficient), then the trend (expected *vs*. non-expected) and statistical signification was evaluated (*p* < 0.05). From the metrics considered redundant, the one with the highest sensitivity, highest correlation coefficient with environmental variables, and the most user-friendly characteristics was selected.

In order to assess relationships between all candidate metrics and environmental variables, including physicochemical and biological predictors, a Redundancy Analysis (RDA) was run using CANOCO [38]. RDA was chosen because previous inspection of the data revealed a linear mode rather than a unimodal response in the biotic variables [39]. Environmental variables that were strongly intercorrelated with others (those with an inflation factor >20) in the initial analysis were excluded. The forward selection option provided by CANOCO was applied and those variables with *p* < 0.05 (Monte Carlo permutation test with 9999 permutations) were kept for the analysis. The final RDA model was run using a set of independent and significant environmental variables [39].

## Results

### Environmental variation of wetlands

The 30 mallines were dissimilar in size but were equally distributed across the three different grazing intensities (Table 2). Most of the water bodies (25) were shallow (average depth <0.6 m). This was reflected in the water temperature which varied from 10.5°C (PQ1) to 25.5°C (SAN). Dissolved oxygen varied from 5.6 mg/L to 18.27 mg/L. However, neither mean temperature nor dissolved oxygen differed significantly among groups (*p* > 0.05).

**Table 2:** Physicochemical and biological features measured at 30 Patagonian wetlands (Argentina), with three levels of livestock grazing impacts. Median, standard deviation and between-brackets minimum and maximum values are consigned per impact level. Superscripts denote Kruskal Willis correlations with p <0.05.

	Livestock grazing impact		
	Low (8 sites)	Medium (15 sites)	High (7 sites)
**Environmental features**			
Precipitation (mm/year)	532.2±222^***a***^(165.2–794.6)	358.8±215.8^***ab***^(120.7–935.8)	204.3±101.5^***b***^(87.4–362.3)
Altitude (m.a.s.l.)	696.4±77.8 (586–808)	719.0±89.2(594–918)	651.7±148.2(448–808)
Water temperature (°C)	15.5±3.3 (10.9–21)	15.9±4.3 (10.5–26.5)	19.1±3.7 (14.1–24.7)
Average depth (cm)	91.4±160.8(13.7–488)	93.1±163.1 (14.2–640)	39.1±49.7 (3.8–150)
Width (m)	71.875±162.8 (3–473)	206.1±481.1 (2–1763)	242.4±557 (2–1500)
Length (m)	230.6±505.5 (8–1457)	370.5±658.3 (5–2146)	357.4±701.2 (2–1900)
Area (ha)	8.2±22.6 (0.002–64.1)	24.8±70.6 (0.001–272.4)	32.8±84.2(0.0003–223.6)
pH	7.2±0.4^***a***^(6.79–8.01)	8.1±0.8^***b***^(6.9–9.1)	8.7±0.5^***c***^(7.99–9.45)
Conductivity (μS/cm)	117.3±130.7^***a***^(28–394)	237.8±158.3^***b***^(61.4–575)	2101±2380.3^***c***^(185–6610)
TDS (mg/L)	65.6±68.1^***a***^(17.8–200)	143.8±104.7^***b***^(37.9–377)	1009.78±1555.3^***c***^(133–4430)
Salinity ‰	0.06±0.07^***a***^(0–0.2)	0.1±0.1^***a***^(0–0.4)	1.4±1.6^***b***^(0.2–4.6)
Dissolved oxygen (mg/L)	10.1±2.8(5.6–13.3)	10.9±2.5(7.7–18.3)	10.5±1.6(9.15–12.8)
Oxygen %	113.3±20.3 (83.1–139)	114±35.5 (84–224)	116.2±22.2 (91–146)
TN (μg/L)	382.2±205.6^***a***^(153–662)	526.9±265.3^***a***^(176–1063)	3437.6±3682^***b***^(504–10514)
NO_2_^–^+NO_3_^–^ (μg/L)	6±10.1(2.5–31)	32.8±86(2.5–316)	4.6±4.3 (2.5–14)
NH_4_^+^ (μg/L)	6.45±3.7^***a***^(4–14)	21.1±28.3^***a***^(4–108)	435±817.6^***b***^(4–2269)
TP (μg/L)	43.6±25.2^*a*^(22–100)	53.7±28.1^*a*^(17–129)	1067.4±1436.8^*b*^(23–3922)
SRP (μg/L)	2.1±1. 4^***a***^(1–5)	10.4±13.4^***a***^(1–41)	643.3±1117.1^***b***^(3–3062)
TN/TP	10.2±7.0(4.3–23.6)	11.3±6.7(2.4–31.1)	8.8±7.3(1.3–21.9)
**Aquatic plants**			
Species richness	6.2±1.9 (4–9)	4.5±2.5(1–10)	4±2.6(1–9)
Exotic richness	1.2±1.6(0–4)	0.9±0.9(0–3)	1.1±1.5(0–4)
Native richness	4.3±1.9(1–8)	3.3±2.1(1–8)	2.7±1.4(1–5)
Endemic richness	0.6±0.9(0–2)	0.3±0.5(0–1)	0.1±0.4(0–1)
Coverage	4.6±1.1^***a***^(3–6)	4.5±1.3^***a***^(2–7)	3.3±0.76^***b***^(2–4)

Physicochemical parameters that significantly varied among site groups were water conductivity, pH, salinity, and total dissolved solids (Kruskal Wallis, *p* < 0.001). An important gradient in pH values was found, circumneutral at low impact sites (6.79) to extremely alkaline at medium and high impact sites (9.45). However, most mallines showed slightly alkaline values (~ 8). Mean conductivity was 117.3, 237.8 and 2101 μS/cm at low, medium and high disturbance sites respectively. Furthermore, the most disturbed wetlands showed conductivity values significantly higher than those with medium or low grazing intensity (Table 2, Kruskal Wallis, *p* < 0.005). Salinity was similar between low (0.06‰) and middle (0.1‰) grazing intensity groups, but these differed from high grazing intensity groups (1.4‰).

Nutrient values were markedly different when contrasting sites at low and medium intensity of grazing with those that were highly grazed. High values of total nitrogen (TN; 4761, 5181 and 10514 μg/L) were recorded at PiP, LaC and DoJ mallines respectively, and these sites also showed the most extreme values of ammonium (Table 2). However, at most wetlands, very low values of nitrates (NO_3_^−2^; 2.5 ≤ μg/L, *n* = 23) and total phosphorus (TP; <130 μg/L, *n* = 26) were observed. Higher values of total phosphorus were also documented in the most impacted wetlands (e.g. 982, 1951 and 3922 μg/L; GUJ, DoJ and LaC).

Aquatic plant attributes were contrasted among groups of sites. Mean values of plant richness varied between 4 and 6.2 taxa at sites with high and low grazing intensity respectively. Coincidently, richness of native plants was 2.7 and 4.3 taxa, in the same two site groups. Plant coverage changed significantly among groups, with highly disturbed sites showing the lowest values (Table 2).

**Fig 2:**
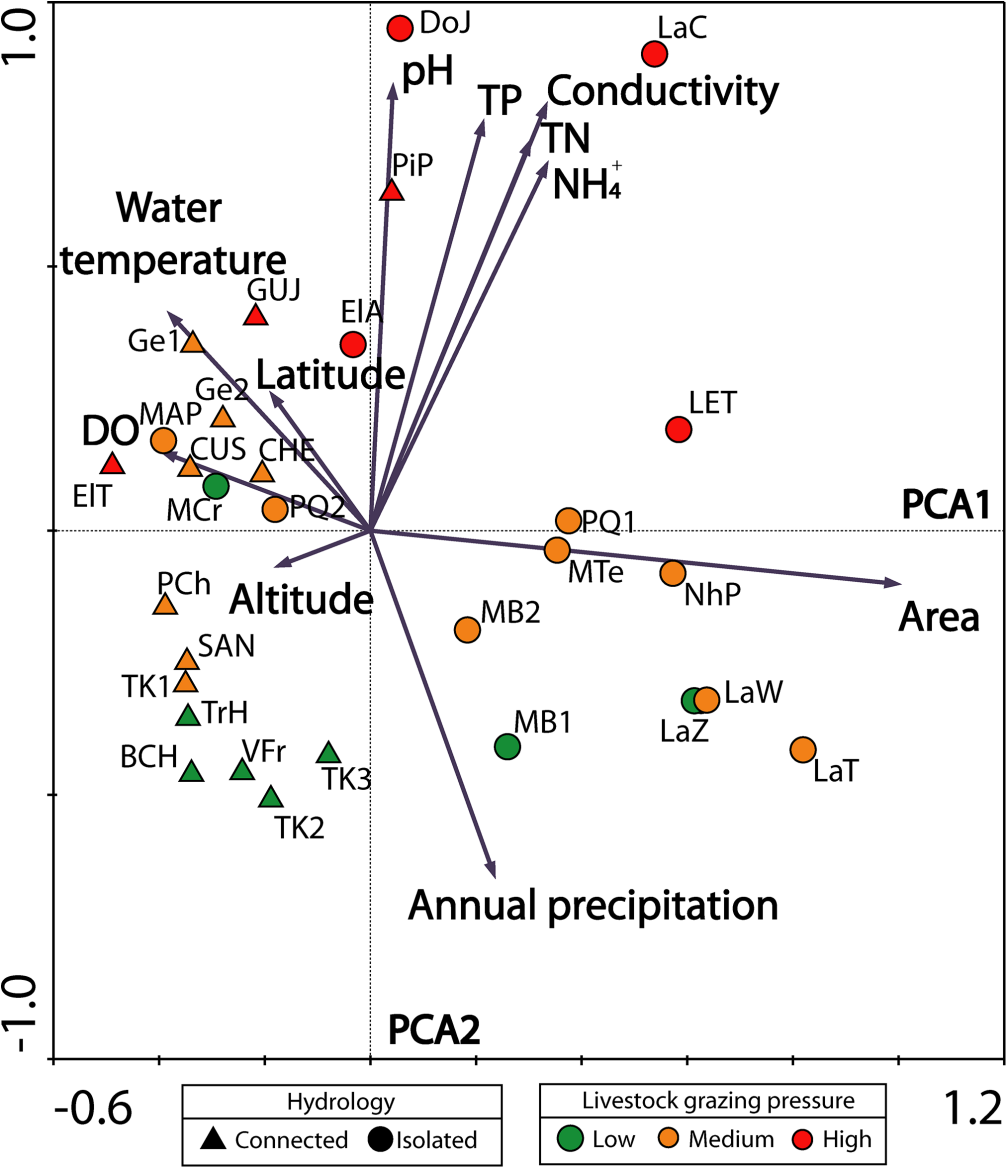
Principal components analysis (PCA) scatterplot based on environmental variables (December 2006 and December 2007) at 30 Patagonian wetlands. Symbols represent connected or isolated wetlands; colors indicate the level of grazing pressure.

Principal Component Analysis (PCA) showed a clear site separation according to environmental characteristics (Fig 2). The first factor explained 74.5% of the sites variance and was mainly determined by a pond’s area, water temperature, and dissolved oxygen. Variables secondarily associated with PCA1 were related with wetland location in geographic space (altitude and latitude). The second component (PCA2) captured 16.4% of the variance of site distribution, and was determined by chemical variables (TN, TP, NH_4_^+^, conductivity and pH) and precipitation. Wetlands associated with small lakes (LaT, LaZ, LaW and LET) and those having large flooded areas (NhP, MTe and PQ1) were located to the positive end of PCA 1 (Fig 2). Sites well oxygenated that displayed higher temperature values were grouped on the negative end of PCA1. Also, this component (PCA1) clearly separated sites according to their hydrological condition, with sites corresponding to connected wetlands (triangles) placed on the negative end of PCA1, and those classified as isolated (circles) grouped to the positive end of PCA1.

The PCA2 axis highlighted the existence of a disturbance gradient, and was explained by the nutrient concentrations (TP, TN, NH_4_^+^), conductivity, and pH. Accordingly, a set of sites (DoJ, LaC and PiP) having nutrient loads and alkaline waters were located on the positive end of PCA2. Grouped on the opposite PCA2 end were sites: SAN, TK1, TK2, TK3, TrH, BCH, VFr, MB1 and MB2. These sites displayed poor nutrient levels and low conductivity. The axis also placed sites along a gradient of rainfall, with sites from more humid areas located in the two lower quadrants. The multivariate analysis allowed us to validate the levels of grazing pressure assigned, with more highly impacted sites placed on the right upper quadrant and lesser impacted sites in the lower left quadrant (Fig 2).

### Metric selection

**Fig 3:**
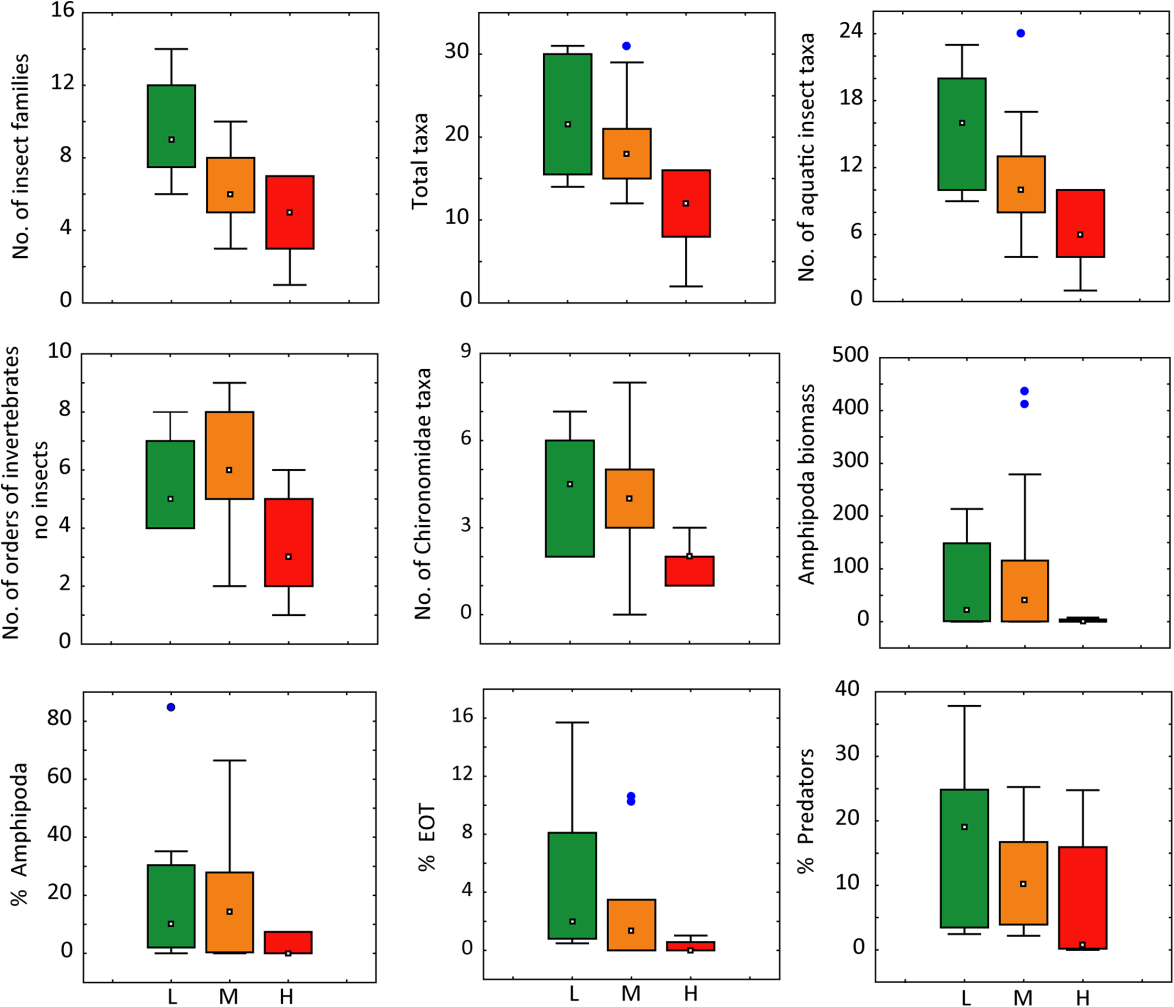
Distribution of values for candidate invertebrate metrics at Patagonia wetlands within three levels of livestock grazing pressure (Argentina), December 2006 and December 2007. L = low; M = medium; H = High. Range bars show maximum and minimum of non-outliers data; boxes are interquartile ranges (25%ile to 75%ile); small squares are medians; dots correspond to outliers.

As a result of the application of the criteria previously defined for all candidate metrics (88), nine were selected to evaluate disturbances in the wetlands (Fig 3). A group of six measures of richness, abundance and biomass (no. of insect families, total taxa, no. of aquatic insect taxa, no. of orders of invertebrates no insects, no. of Chironomidae taxa and Amphipoda biomass), two of composition (% Amphipoda and % EOT) and one of functional feeding groups (% predators), displayed the best performance.

The metric no. of insect families showed consistent, strong sensitivity (type 3), with median values of 9 and 5 families at sites with low and high levels of disturbance, respectively. The remaining metrics of richness, abundance and biomass, showed the same behavior, but were not as consistent, being more variable at lesser grazed sites (Fig 3).

The metric n° orders of invertebrates no insects, showed the highest values in middle grazed sites, and was lowest in the highly disturbed wetlands. However, it was retained because it is easy to calculate (low effort of taxonomic resolution), and could improve the discrimination power if the number of reference sites increase. The other three metrics (% Amphipoda, % EOT, and % predators) showed great variability at reference sites; nevertheless, they displayed strong sensitivity to disturbance.

Covariation assessed within metrics based on richness, abundance, and biomass data revealed that some measures were redundant (r > 0.70). For example, no. of aquatic insect taxa and total taxa were strongly correlated with the no. of insect families and number of Chironomidae taxa (r > 0.78). These two metrics provided the same information that the no. of insect families, therefore they were discarded in favor of the no. of insect families. On the other hand, % predators was correlated with % EOT (r = 0.76), but both were preserved as they provided different information about the aquatic invertebrate community.

### Water quality features, relationships with metrics

**Fig 4:**
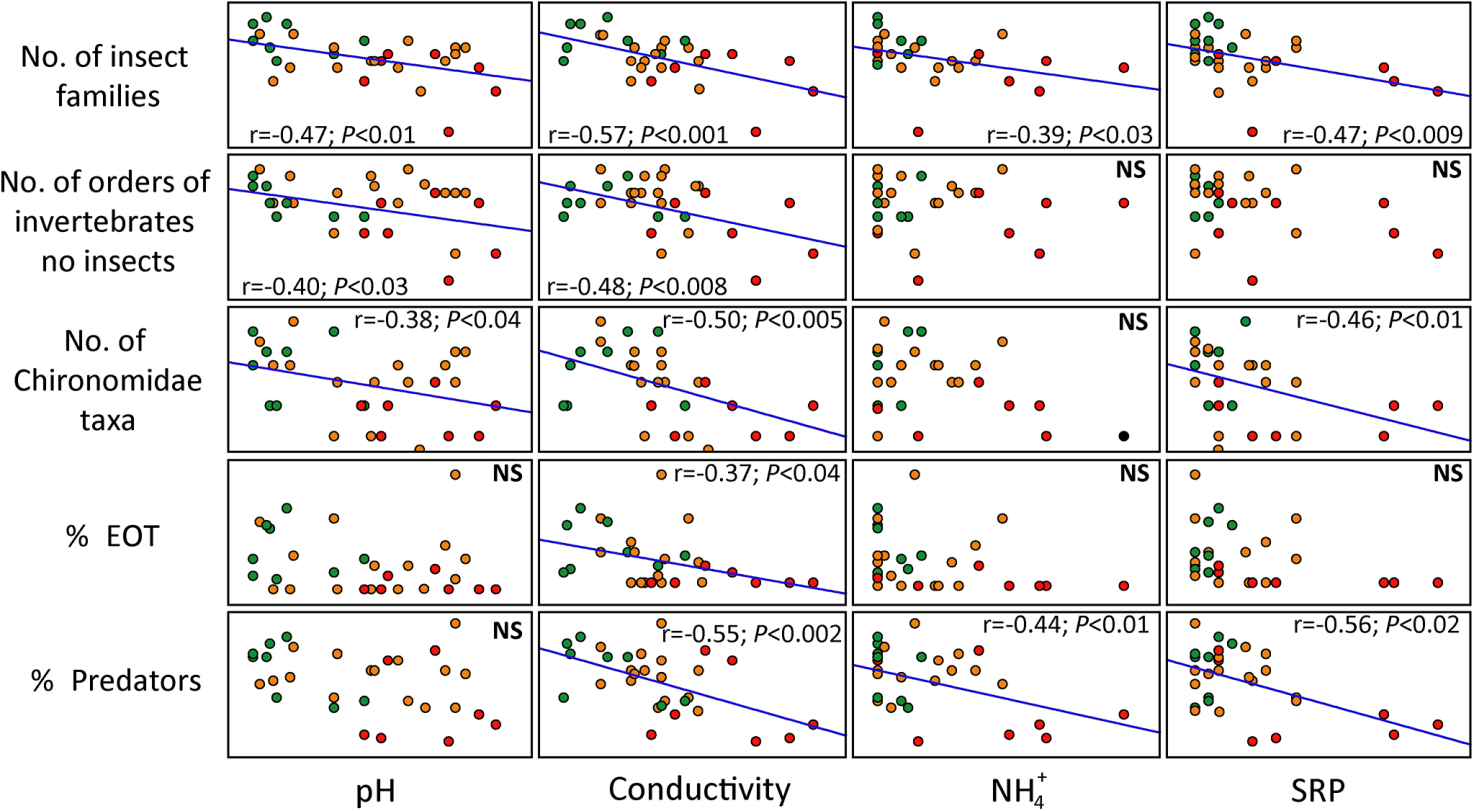
Scatterplot matrix of five evaluated metrics, including Pearson correlation coefficient and statistical signification (NS: non-significant). A 1:1 line has been added to graphics to depict the relationship between metrics and the four environmental features. SRP: soluble reactive phosphorous, NH_4_: ammonium. Symbols represent grazing intensity as follows: red: high, orange: medium and green: low.

Several significant and predicted responses were observed between selected metrics and water quality variables (Fig 4). None of the selected metrics varied significantly with annual rainfall, water temperature, mean depth, area of the water body, dissolved oxygen, or total richness, of aquatic plants (*P* > 0.05). With the exception of % Amphipoda and Amphipoda biomass, all selected metrics showed at least one significant correlation with water quality related parameters (pH, conductivity, and nutrients) (Fig 4).The expected response of metrics was a decrease in values as nutrient, pH, and conductivity values increased. Particularly, no. of insect families and n° of Chironomidae taxa decreased significantly with the rise of nutrients (TN, TP and SRP). Moreover, no. of insect families and % predators decreased significantly with NH_4_^+^ increases.

### Multivariate analysis

**Fig 5:**
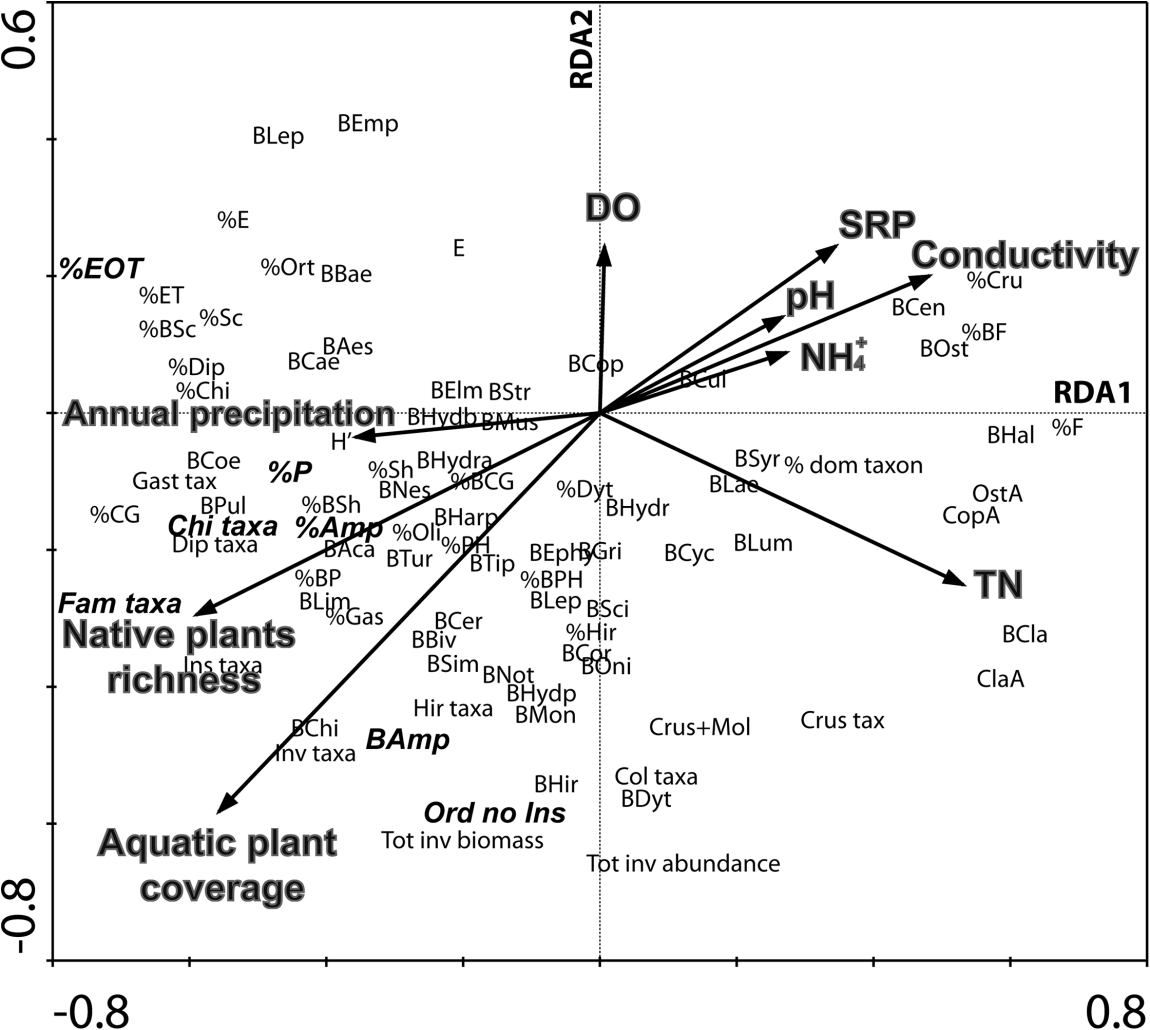
RDA ordination diagram based on environmental relationships of 88 invertebrate metrics at 30 Patagonian wetlands (December 2006 and December 2007). Seven selected metrics are in bold letters. Metric codes are in S1 Table.

The RDA ordination revealed a strong relationship between invertebrate metrics and environmental variables (Table 3), with correlation coefficients of 0.86 and 0.70 for the first and second axis, respectively. Conductivity, TN, aquatic native plant richness, and aquatic plant coverage were the most significant variables explaining variation in the 88 candidate metrics (Table 3; Fig 5). The first and the second axes explained 57.3% of the total variation in the metrics-environmental relationships. The first axis (F = 3.61, *p*< 0.008) and the overall model (F = 1.603, *p*< 0.002) were statistically significant. The disturbance gradient (RDA 1) was mostly determined by conductivity, TN, the richness of aquatic native plants and the aquatic plant coverage. The 7 selected metrics were associated to the negative endpoint of this axis (Fig 5).

**Table 3:** RDA results for 30 Patagonian wetlands of Chubut Province, Patagonia, Argentina. Eigenvalues and correlation of standardized environmental variables with the first two RDA axes of each analysis is consigned. *F*-ratio statistics are listed for the first axis and for all the axes combined.

Variable	Axis	
	RDA1	RDA2
pH	0.26	0.14
Conductivity	**0.48**	0.20
DO	0.01	0.24
TN	**0.53**	-0.25
NH_4_^+^	0.27	0.09
SRP	0.35	0.24
Precipitation	-0.36	-0.03
Aquatic native plants richness	**-0.59**	-0.30
Aquatic plant coverage	**-0.56**	**-0.58**
Eigenvalues	0.153	0.087
Species-environment correlation	0.86	0.70
Cumulative percentage variance		
of species data	15.3	24.0
of species-environment relation	36.5	57.3
*p*–values for Monte Carlo Permutation test		
Axis 1: F = 3.61, *p*< 0.008		
All canonical axes: F = 1.603, *p*< 0.002		

## Discussion

### Environmental background of Patagonian wetlands

This research identified significant changes in physicochemical features (mainly pH, conductivity, and nutrient values) across the gradient of the 30 studied Patagonian wetlands. While, the set of mallines included small isolated sites, lacunar systems and wetlands associated with rivers and streams (connected), the variation of environmental parameters fitted well with the identified levels of grazing intensity, and this disturbance gradient was validated by the multivariate analysis. Wetlands impacted by farming activities and pasture improvement usually have higher levels of nitrogen, phosphorus, turbidity, conductivity, and pH than undisturbed sites [40–42], indicating that these trends are strongly related to anthropogenic actions. However, the variation of certain physicochemical characteristics (e.g. salinity, conductivity, pH) could respond to both natural and human causes, which is expected when analyzing wide environmental gradients such as we describe here. The most highly impacted wetlands were generally located at the East, which was expected since water scarcity forces landowners to increase livestock density in wetlands. Hence, water conductivity should be related to the nature and origin of soils, as well as to annual rainfall; however, others have shown that overgrazing has also promoted the erosion and salinization of several Patagonian environments including wetlands [43]. Finally, the variation of conductivity can be explained at least in part by the current land use practice.

Regardless of the value of annual average precipitation, symptoms of eutrophication were remarkable at some sites in the form of high nutrient concentrations. At least four of the more highly disturbed wetlands displayed extreme concentrations of nitrogen and total phosphorus suggesting hypertrophy [44], presumably caused by the presence of livestock. Moreover, nutrient values were recorded that exceeded those documented by other authors in grazed wetlands [7], including some in highly disturbed areas [41,45]. Nutrient enrichment in wetlands promotes changes in the composition and abundance of algae and macrophytes, sometimes by reducing their species richness and increasing the abundance of exotic species [41]. Plants are considered key habitat components in aquatic environments for their role in increasing habitat complexity [46], and for their importance as a food source [47]. In this study, the metrics redundancy analysis revealed that an increase in nutrients and loss or diminution of the richness of native aquatic plants can have detrimental effects on the biomass, abundance, and richness of invertebrate.

### Invertebrate metrics

Seven invertebrate metrics consistently and accurately described the effects of livestock grazing on wetlands. This set of metrics included measures of richness, abundance, biomass, composition and feeding groups, representing different communities attributes. In agreement with other reports from the United States [48] and Spain [49], these metrics were negatively correlated with increased levels of grazing disturbance. In addition, they responded as expected to variation of water quality parameters (nutrients, pH, and conductivity).

The number of insect families was the most robust metric. Taxonomic resolution to genus or species level often gives accurate results and provides more information, but it requires more training, adequate knowledge, and more time [16]. As observed by other authors [13], information at the family level may reflect community patterns that are similar to those obtained with more refined taxonomic information, and thus can be considered appropriate to use in the analysis of the status of Patagonian wetlands.

The % EOT proved to be significantly sensitive to the increase of water conductivity, but also decreased with increasing pH and nutrient levels. This metric has been successfully used in other studies [16], being sensitive to chemical factors and anthropogenic disturbances (e.g. increased turbidity [50]) and was, therefore, expected to decrease with increased livestock intensity. As expected, the % predators decreased with increasing disturbance. Invertebrate predator assemblages are usually good predictors of overall aquatic invertebrate community richness. Others have found that by modifying the structure of the natural plant community, livestock grazing can negatively affect the abundance of predators [51].

In temperate areas, aquatic invertebrates have been demonstrated to be good biological indicators of wetland ecological health [52,53]. Our multivariate approach revealed that the 88 metrics responded consistently to five key environmental variables: pH, conductivity, dissolved oxygen, nutrients, and aquatic plant coverage and richness. Similar results have been reported by other authors [15,54]. The strongest responses involved the positive relationships between invertebrate metrics and aquatic plant coverage and native species richness. We propose the following explanation: wetlands are structurally complex habitats [55], and the disturbances caused by grazing tend to reduce their complexity by affecting the richness and coverage of aquatic plants. Livestock remove substantial amounts of vegetation and trample the ground, effectively reducing substrate and shelter availability thereby affecting invertebrate community structure. As demonstrated recently by Kutschker *et al*. [3], there was significantly lower richness of macrophytes at heavily grazed sites compared to those having low grazing pressure.

The metrics Shannon-Weaver diversity and % dominant taxon have been frequently used as measures of rapid assessment of wetlands health [35,41]. As observed in the RDA analysis, both metrics could function properly for assessing Patagonian wetland quality but should be tested by adding a higher number of study sites.

### Wetlands management and conservation strategies

The physical effects most markedly associated with livestock included high feces concentrations; soil erosion and salinization; and signs of foraging and trampling. Different agencies such as INTA (e.g. [56]) have suggested that mallines should be fenced in order to assign a particular function to them in rodeo management (e.g. lamb fattening). This practice has also been recommended for Australian wetlands [57], with good results. However, this conservation action was rarely documented at sites covered by this study. Proposed conservation recommendations originating in other countries that might also be effective in Patagonia include the creation of artificial ponds and mechanical troughs for livestock watering [58]. Due to the fact that livestock producers are ultimately the ones who decide how to manage Patagonian fields, it would be desirable to alert them to the consequences of the livestock grazing practices on wetland performance and quality. Knowledge of wetland trophic structure, richness, and biodiversity may be relevant not only for natural resource managers, but also for landowners. This work enriched interactions between concerned parties, with government institutions (such as INTA) now receiving farmers’ feedback, and providing farmers with quality information in return.

### Final considerations

In this study we defined three levels of grazing intensity which were easily measured in the field by combining information related to eight features of thirty Patagonian wetlands. Not only are these metrics easy to calculate, but they provide accurate information about the wetland’s conservation status and could also roughly reflect the water nutrient levels.

The most prominent metric is "number of insect families"; indicating that, with a quick training in insect family identification, this metric could be used to rapidly assess the ecological condition of the ponds of NW Patagonia. However, in addition to this and other metrics, we also recommend that the physicochemical measurements of the ponds be taken, since these data could be used to distinguish between natural and anthropogenic effects.

We suggest the inclusion of aquatic invertebrate metrics in future monitoring programs, given their sensitivity to different levels of livestock impacts, low cost, easy sampling, and their relevance to complement the information provided by other biological quality elements frequently used in such programs worldwide. It would also be desirable that future conservation actions include Patagonian ponds as part of a mosaic of freshwater habitats.

The results of this study reveal that ponds from arid and semi-arid Patagonia support high invertebrate community biodiversity, yet they are vulnerable to livestock grazing practices. Unfortunately, arid and semi-arid Patagonia have very few protected areas. Moreover, pond monitoring or restoration programs are rare; therefore this study can be considered as the first step for the use of aquatic invertebrates as potential candidates for biomonitoring in Patagonia.

## Supporting Information

S1 Table Invertebrate metrics data. Quartiles of macroinvertebrate metrics from 30 ponds at Patagonian wetlands. Data sets correspond to different categories of grazing intensity: low, medium and high. Seven candidate metrics are in bold. Codes for RDA are also displayed.
(DOCX)

## References

[1] Hansson LA, Brönmark C, Nilsson PA, Åbjörnsson K. Conflicting demands on wetland ecosystem services: Nutrient retention, biodiversity or both? Freshwater Biol 2005; 50: 705–714.

[2] Semlitsch RD, Peterman WE, Anderson TL, Drake DL, Ousterhout BH. Intermediate Pond Sizes Contain the Highest Density, Richness, and Diversity of Pond-Breeding Amphibians. PLoS ONE 2015; 10: e0123055 doi: 10.1371/journal.pone.0123055 25906355 10.1371/journal.pone.0123055 PMC4408075

[3] Kutschker AM, Epele LB, Miserendino ML. Aquatic plant composition and environmental relationships in grazed Northwest Patagonian wetlands, Argentina. Ecol Eng 2014; 64: 37–48.

[4] Perotti MG, Diéguez MC, Jara FG. Estado del conocimiento de humedales del norte patagónico (Argentina): aspectos relevantes e importancia para la conservación de la biodiversidad regional. Rev Chil Hist Nat 2005; 78: 723–737.

[5] Epele LB, Archangelsky M. Spatial variation of water beetle communities in arid and semi-arid Patagonian wetlands and their value as environmental indicators. Zool Stud 2012; 51: 1418–1431.

[6] Mazzoni E, Vázquez M. Ecosistemas de mallines y paisajes de la Patagonia Austral (Provincia de Santa Cruz). INTA; 2004.

[7] Schmutzer AC, Gray MJ, Burton EC, Miller DL. Impacts of cattle on amphibian larvae and the aquatic environment. Freshwater Biol 2008; 53: 2613–2625.

[8] Gaitán JJ, López CR, Bran D. Vegetation composition and its relationship with the environment in mallines of north Patagonia, Argentina. Wetl Ecol Manag 2011; 19: 121–130.

[9] Kepner WG, Watts CJ, Edmonds CM, Maingi JK, Marsh SE, Luna G. A landscape approach for detecting and evaluating change in a semi-arid environment. Environ Monit Assess 2000; 64: 179–195.

[10] del Valle HF, Elissalde NO, Gagliardini DA, Milovich J. Status of desertification in the Patagonian region: assessment and mapping from satellite imagery. Arid Soil Res Rehab 1998; 12: 1–27.

[11] Masiokas MH, Villalba R, Luckman BH, Lascano ME, Delgado S, Stepanek P. 20th-century glacier recession and regional hydroclimatic changes in northwestern Patagonia. Global Planet Change 2008; 60: 85–100.

[12] Crego RD, Nielsen CK, Didier K. Climate change and conservation implications for wet meadows in dry Patagonia. Environ Conserv 2014; 41: 122–131.

[13] Balcombe CK, Anderson JT, Fortney RH, Kordek WS. Aquatic macroinvertebrate assemblages in mitigated and natural wetlands. Hydrobiologia 2005; 541: 175–188.

[14] Sharma RC, Rawat JS. Monitoring of aquatic macroinvertebrates as bioindicator for assessing the health of wetlands: A case study in the Central Himalayas, India. Ecol Indic 2009; 9: 118–128.

[15] Mereta ST, Boets P, De Meester L, Goethals PLM. Development of a multimetric index based on benthic macroinvertebrates for the assessment of natural wetlands in Southwest Ethiopia. Ecol Indic 2013; 29: 510–521.

[16] U.S. EPA. Methods for Evaluating Wetland Condition: Developing an Invertebrate Index of Biological Integrity for Wetlands. DC. EPA-822-R-02-019. Washington: Office of Water, U.S. Environmental Protection Agency; 2002.

[17] Wissinger SA. Ecology of wetland invertebrates: synthesis and application to wetlands management In: Batzer DP, Rader RB, Wissinger SA, editors. Invertebrates in freshwater wetlands of North America: ecology and management. New York: Wiley; 1999 p. 1043–1086.

[18] Miserendino ML, Brand C, Di Prinzio CY. Assessing urban impacts on water quality, benthic communities and fish in streams of the Andes Mountains, Patagonia (Argentina). Water Air Soil Poll 2008; 194: 91–110.

[19] Tell G, Izaguirre I, Quintana, R. Flora y Fauna Patagónicas. Ediciones Caleuche. Bariloche; 1997.

[20] Instituto Nacional de Tecnología Agropecuaria (INTA) [Internet]. Meteorological data. Available: http://inta.gob.ar/documentos/datos-meteorologicos-de-eea-esquel. Accessed 15 May 2015.

[21] Epele LB, Miserendino ML. Temporal dynamic of invertebrate and aquatic plant communities at three intermittent ponds in livestock grazed Patagonian wetlands. J Nat Hist 2015, 8 22 doi: 10.1080/00222933.2015.1062930

[22] Casamiquela R. El poblamiento de la Patagonia. Confines digital; 2007.

[23] Siffredi GL, López CR, Bran DE, Ayesa JA, Gaitán JJ, Becker GF. Guía de recomendación de carga animal para mallines INTA EEA Bariloche-“Dr. Grenville Morris”; 2007.

[24] Mitsch WJ, Gosselink JG. Wetlands. New York: John Wiley & Sons, Inc; 2007.

[25] Jobbágy EG, Paruelo JM, León RJC. Estimación del régimen de precipitación a partir de la distancia a la cordillera en el noroeste de la Patagonia. Ecología Austral 1995; 5: 47–53.

[26] Quirós R. Relationships between air temperature, depth, nutrients and chlorophyll in 103 Argentinean lakes. Verh InternatVerein Limnol 1988; 23; 647–658.

[27] American Public Health Association (APHA). Standard Methods for the examination of water and wastewater Maryland USA: American Water Works Association, Hanover; 1994.

[28] Correa MN. Flora Patagónica. Vol. VIII: part I, II, III, IVa, IVb, V, and VIII; 1978–1999.

[29] Galatowitsch SM, Whited DC, Lehtinen R, Husveth J, Schik K. The vegetation of wet meadows in relation to their land-use. Environ Monit Asses 2000; 60: 121–144.

[30] Domínguez E, Fernández HR. Macroinvertebrados bentónicos sudamericanos Sistemática y biología. Fundación Miguel Lillo, Tucumán, Argentina; 2009.

[31] Benke AC, Huryn AD, Smock LA, Wallace JB. Length-mass relationships for freshwater macroinvertebrates in North America with particular reference to the southeastern United States. J N Am Benthol Soc 1999; 18: 308–343.

[32] Epele LB, Miserendino ML. Life cycle, production and habitat selection of *Notoperla fasciata* and *N*. *magnaspina* (Plecoptera: Gripopterygidae) in a headwater Patagonian stream. Fund Appl Limnol 2011; 178: 219–229.

[33] Merritt RW, Cummins KW, Berg MB. Aquatic Insects of North America. Iowa: Kendall/Hunt Publishing Company; 2008.

[34] Barbour AMT, Gerritsen J, Griffith GE, Frydenborg R, McCarron E, White JS, et al A framework for biological criteria for Florida streams using benthic macroinvertebrates. J N Am Benthol Soc 1996; 15: 185–211.

[35] Tangen BA, Butler MG, Ell M. Weak correspondence between macroinvertebrate assemblages and land use in prairie pothole region wetlands USA. Wetlands 2003; 23: 104–115.

[36] Maloney KO, Feminella JW. Evaluation of single and multi-metric benthic macroinvertebrate indicators of catchment disturbance over time at the Fort Benning Military Installation, Georgia, USA. Ecol Indic 2006; 6: 469–484.

[37] Ludwing JA, Reynolds JF. Statistical Ecology. New York: Wiley; 1988.

[38] ter Braak CJF, Smilauer P. CANOCO for Windows (vers, 4.02) A FORTRAN program for canonical community ordination. Wageningen: Wageningen, the Netherlands: Centre for Biometry; 1999.

[39] ter Braak CJF, Smilauer P. CANOCO reference manual and users guide to Canoco for Windows Wageningen: Wageningen, the Netherlands: Centre of Biometry; 1998.

[40] Gleason RA, Euliss NH, Hubbard DE, Duffy WE. Effects of sediment load on emergence of aquatic invertebrates and plants from wetland soil egg and seed banks. Wetlands 2003; 23: 26–34.

[41] Chipps SR, Hubbard DE, Werlin KB, Haugerud NJ, Powell KA, Thompson J. Association between wetland disturbance and biological attributes in floodplain wetlands. Wetlands 2006; 26: 497–508.

[42] Bornette G, Puijalon S. Response of aquatic plants to abiotic factors: a review. Aquat Sci 2011; 73: 1–14.

[43] Collantes MB, Faggi AM. Los humedales del sur de Sudamérica In: Málvarez AI, editor. Tópicos sobre humedales subtropicales y templados de Sudamérica. Montevideo, Uruguay: UNESCO; 1999 p. 15–25.

[44] OECD. Eutrophication of waters Monitoring, assessment and co*ntrol*. Paris: OECD; 1982.

[45] Steinman AD, Conklin J, Bohlen P, Uzarski DG. Influence of cattle grazing and pasture land use on macroinvertebrate communities in freshwater wetlands. Wetlands 2003; 23: 877–889.

[46] Poi de Neiff AS, Casco SL. Biological agents that accelerate winter decay of *Eichhornia crassipes* Mart. Solms. in northeastern Argentina In: Thomaz SM, Bini LM, editors. Ecologia e Manejo de Macrófitas Aquáticas. Maringa: Eduem; 2003 p. 127–144.

[47] Bazzanti M, Coccia C, Giuseppina M. Microdistribution of macroinvertebrates in a temporary pond of Central Italy : Taxonomic and functional analyses. Limnologica 2010; 40: 291–299.

[48] Barbour AMT, Stribling JB, Karr JR. The multimetric approach for establishing biocriteria and measuring biological condition In: Davis WS, Simon TP editors. Biological assessment and criteria: tools for water resource planning and decision making. Florida: Lewis Publishers, Boca Raton; 1995 p. 63–80.

[49] Boix D, Gascón S, Badosa A, Brucet S, López-Flores R, Martinoy M. Patterns of composition and species richness of crustaceans and aquatic insects along environmental gradients in Mediterranean water bodies. Hydrobiologia 2008; 597: 53–69.

[50] Stewart TW, Downing JA. Macroinvertebrate communities and environmental conditions in recently constructed wetlands. Wetlands 2008; 28: 141–150.

[51] Foote L, Rice Hornung CL. Odonates as biological indicators of grazing effects on Canadian prairie wetlands. Ecol Entomol 2005; 30: 273–283.

[52] Batzer DP. The Seemingly Intractable Ecological Responses of Invertebrates in North American Wetlands: A Review. Wetlands 2013; 33: 1–15.

[53] Ilmonen J, Virtanen R, Paasivirta L, Muotka T. Detecting restoration impacts in inter-connected habitats: Spring invertebrate communities in a restored wetland. Ecol Indic 2013; 30: 165–169.

[54] Menetrey N, Sager L, Oertli B, Lachavanne JB. Looking for metrics to assess the trophic state of ponds. Macroinvertebrates and amphibians. Aquat Conserv 2005; 15: 653–664.

[55] Biggs J, Williams P, Whitfield M, Nicolet P, Weatherby A. 15 years of pond assessment in Britain: results and lessons learned from the work of Pond Conservation. Aquat Conserv 2005; 15: 693–714.

[56] Nakamatsu V. Pastoreo de mallines: cálculo de la capacidad de carga. INTA Ganadería 2006; 20: 87–92.

[57] Davies KF, Melbourne BA, James CD, Cunningham RB. Using traits of species to understand responses to land use change: Birds and livestock grazing in the Australian arid zone. Biol Conserv 2010; 143: 78–85.

[58] Declerck S, De Bie T, Ercken D, Hampel H, Schrijvers S, Van Wichelen J, et al Ecological characteristics of small farmland ponds: Associations with land use practices at multiple spatial scales. Biol Conserv 2006; 131: 523–532.

