# Supplementary material for: Environmental Quality and Aquatic Invertebrate Metrics Relationships at Patagonian Wetlands Subjected to Livestock Grazing Pressures
Source: PLoS One. 2015 Oct 8;10(10):e0137873. doi: 10.1371/journal.pone.0137873 (PMC4598092; doi:10.1371/journal.pone.0137873)
Supplement: S1 Table — Quartiles of macroinvertebrate metrics from 30 ponds at Patagonian wetlands. Data sets correspond to different categories of grazing intensity: low, medium and high. Seven candidate metrics are in bold. Codes for RDA are also displayed. (DOCX) [file pone.0137873.s001.docx]

**Invertebrate metrics data**

S1 Table. Quartiles of macroinvertebrate metrics from 30 ponds at Patagonian wetlands. Data sets correspond to different categories of grazing intensity: low, medium and high. Seven candidate metrics are in bold. Codes for RDA are also displayed.

| **Metric** | **Code for RDA** | **Low** | | | **Medium** | | |  | **High** | | | |
| --- | --- | --- | --- | --- | --- | --- | --- | --- | --- | --- | --- | --- |
|  |  | **25th**  **%ile** | **Median** | **75th**  **%ile** | **25th**  **%ile** | **Median** | **75th**  **%ile** | | **25th**  **%ile** | **Median** | **75th**  **%ile** |  |
| Total taxa | Inv taxa | 15.8 | 21.5 | 30 | 15.5 | 18 | 21 | | 8.5 | 12 | 14.5 |  |
| **No. of insect families** | **Fam taxa** | **7.8** | **9** | **12** | **5** | **6** | **8** | | **3.5** | **5** | **6.5** |  |
| **No. of orders of invertebrates no insects** | **Ord no Ins** | **4** | **5** | **7** | **5** | **6** | **7.5** | | **2.5** | **3** | **5** |  |
| No. of aquatic insect taxa | Ins taxa | 10 | 16 | 20 | 8 | 10 | 12.5 | | 5 | 6 | 10 |  |
| No. of Crustacea taxa | Crus taxa | 2.8 | 3 | 3.3 | 2.5 | 4 | 5 | | 2 | 2 | 3.5 |  |
| No. of Crustacea + Mollusca taxa | Crus+Mol taxa | 3 | 3.5 | 5 | 3.5 | 5 | 6 | | 2 | 2 | 3.5 |  |
| No. of Gastropoda taxa | Gast taxa | 0 | 0.5 | 1 | 0 | 0 | 1 | | 0 | 0 | 0 |  |
| No. of Hirudinea taxa | Hir taxa | 0 | 0.5 | 2 | 0.5 | 1 | 2.5 | | 0 | 0 | 0.5 |  |
| No. of Coleoptera taxa | Col taxa | 2.8 | 5.5 | 7 | 2 | 3 | 4.5 | | 2 | 3 | 4 |  |
| **No. of Chironomidae taxa** | **Chi taxa** | **2** | **4.5** | **5.5** | **3** | **4** | **4.5** | | **1** | **2** | **2** |  |
| No. of Diptera taxa | Dip taxa | 3.8 | 7 | 7 | 3 | 4 | 5 | | 1.5 | 3 | 3 |  |
| Total invertebrate abundance | Tot inv ab | 494.8 | 861.8 | 1164.8 | 365.5 | 727 | 1483.1 | | 145.4 | 286.1 | 2488.3 |  |
| Total invertebrate biomass | Tot inv bio | 149.2 | 264 | 447.5 | 170.2 | 214.3 | 514.4 | | 36 | 108.1 | 320.5 |  |
| Insect family biomass |  |  |  |  |  |  |  | |  |  |  |  |
| Gripopterygidae | BGri | 0 | 0 | 0.3 | 0 | 0 | 0 | | 0 | 0 | 0 |  |
| Baetidae | BBae | 0 | 0 | 0 | 0 | 0 | 0 | | 0 | 0 | 0 |  |
| Caenidae | BCae | 0 | 0 | 0 | 0 | 0 | 0.1 | | 0 | 0 | 0 |  |
| Leptophlebiidae | BLep | 0 | 0 | 0 | 0 | 0 | 0 | | 0 | 0 | 0 |  |
| Nesameletidae | BNes | 0 | 0 | 0 | 0 | 0 | 0 | | 0 | 0 | 0 |  |
| Oniscigastridae | BOni | 0 | 0 | 0 | 0 | 0 | 0 | | 0 | 0 | 0 |  |
| Hydrobiosidae | BHydb | 0 | 0 | 0 | 0 | 0 | 0 | | 0 | 0 | 0 |  |
| Hydroptilidae | BHydp | 0 | 0 | 0 | 0 | 0 | 0 | | 0 | 0 | 0 |  |
| Leptoceridae | BLep | 0 | 0 | 1.8 | 0 | 0 | 0 | | 0 | 0 | 0 |  |
| Limnephilidae | BLim | 1.7 | 4.2 | 6.3 | 0 | 0 | 3.4 | | 0 | 0 | 0 |  |
| Elmidae | BElm | 0 | 0 | 0 | 0 | 0 | 0 | | 0 | 0 | 0 |  |
| Hydrophilidae | BHydr | 2 | 13.8 | 38.3 | 0.8 | 5.6 | 25.3 | | 0.2 | 6.1 | 26.2 |  |
| Dytiscidae | BDyt | 3.5 | 17 | 63.9 | 4.7 | 20.4 | 47.3 | | 0.5 | 15.2 | 49.6 |  |
| Hydraenidae | BHydra | 0 | 0 | 0 | 0 | 0 | 0 | | 0 | 0 | 0 |  |
| Haliplidae | BHal | 0 | 0 | 0 | 0 | 0 | 0 | | 0 | 0 | 0.5 |  |
| Scirtidae | BSci | 0 | 0 | 0.1 | 0 | 0 | 0 | | 0 | 0 | 0 |  |
| Coenagrionidae | BCoe | 0.6 | 3.6 | 64.7 | 0 | 0.8 | 3.5 | | 0 | 0 | 0 |  |
| Aeshnidae | BAes | 0 | 0 | 8.9 | 0 | 0 | 0 | | 0 | 0 | 0 |  |
| Corixidae | BCor | 0.2 | 0.5 | 6.4 | 0.2 | 1.8 | 110.1 | | 0 | 0.4 | 7.2 |  |
| Notonectidae | BNot | 0 | 0 | 1.1 | 0 | 0 | 1.9 | | 0 | 0 | 0 |  |
| Chironomidae | BChi | 0.6 | 8.6 | 17.9 | 2.1 | 7.6 | 10.8 | | 0.4 | 0.8 | 2.1 |  |
| Ceratopogonidae | BCer | 0 | 0 | 0 | 0 | 0 | 0 | | 0 | 0 | 0 |  |
| Culicidae | BCul | 0 | 0 | 0 | 0 | 0 | 0 | | 0 | 0 | 0 |  |
| Empididae | BEmp | 0 | 0 | 0 | 0 | 0 | 0 | | 0 | 0 | 0 |  |
| Ephydridae | BEphy | 0 | 0.1 | 0.4 | 0 | 0 | 0 | | 0 | 0 | 1 |  |
| Muscidae | BMus | 0 | 0 | 0 | 0 | 0 | 0 | | 0 | 0 | 0 |  |
| Simuliidae | BSim | 0 | 0 | 5.7 | 0 | 0 | 0 | | 0 | 0 | 0 |  |
| Stratiomydae | BStr | 0 | 0 | 0 | 0 | 0 | 0 | | 0 | 0 | 0 |  |
| Syrphidae | BSyr | 0 | 0 | 0 | 0 | 0 | 0 | | 0 | 0 | 0 |  |
| Tipulidae | BTip | 0 | 0 | 0 | 0 | 0 | 0 | | 0 | 0 | 0 |  |
| Invertebrate (no insect) order biomass |  |  |  |  |  |  |  | |  |  |  |  |
| Turbellaria | BTur | 0 | 0 | 0.1 | 0 | 0 | 0 | | 0 | 0 | 0 |  |
| Lumbriculidae | BLum | 0 | 0 | 0.5 | 0 | 0 | 0.2 | | 0 | 0 | 0.8 |  |
| Moniligastrida | BMon | 0.5 | 3.7 | 35.6 | 6.3 | 8.5 | 26.6 | | 0 | 0.4 | 13.6 |  |
| Hirudinea | BHir | 0 | 3.8 | 12.5 | 0.8 | 4.5 | 24.2 | | 0 | 0 | 2.3 |  |
| Bivalvia | BBiv | 0 | 0 | 3.4 | 0 | 0 | 0 | | 0 | 0 | 0 |  |
| Pulmonata | BPul | 0 | 1.2 | 3.8 | 0 | 0 | 4.9 | | 0 | 0 | 0 |  |
| Laevicaudata | BLae | 0 | 0 | 0 | 0 | 0 | 0 | | 0 | 0 | 0 |  |
| Cladocera | BCla | 0 | 0 | 0.1 | 0 | 0 | 0.7 | | 0 | 0.2 | 0.4 |  |
| **Amphipoda** | **BAmp** | **1.4** | **21.4** | **138.1** | **0.7** | **40.3** | **101** | | **0** | **0** | **3.9** |  |
| Copepoda | BCop | 0 | 0 | 0 | 0 | 0 | 0 | | 0 | 0 | 0 |  |
| Cyclopoida | BCyc | 0 | 0 | 0 | 0 | 0 | 0 | | 0 | 0 | 0 |  |
| Calanoida | BCen | 0 | 0 | 0 | 0 | 0 | 0 | | 0 | 0 | 0 |  |
| Harpacticoidea | BHarp | 0 | 0 | 0 | 0 | 0 | 0 | | 0 | 0 | 0 |  |
| Ostracoda | BOst | 0 | 0.1 | 21.2 | 0.1 | 0.2 | 7 | | 0 | 0.2 | 17 |  |
| Acari | BAca | 0 | 0 | 1.2 | 0 | 0 | 0.1 | | 0 | 0 | 0 |  |
| Ostracoda abundance | OstA | 1.8 | 4.7 | 67.8 | 5.5 | 14.1 | 57 | | 0 | 9.4 | 73.9 |  |
| Copepoda abundance | CopA | 0 | 0.4 | 1.6 | 0 | 0.8 | 18.9 | | 0 | 0 | 17.7 |  |
| Cladocera abundance | ClaA | 0 | 0 | 14.3 | 0 | 5.5 | 105.3 | | 2.8 | 32.2 | 68.4 |  |
| H' | H' | 1.3 | 2 | 2.2 | 1.7 | 1.9 | 2 | | 0.3 | 1.3 | 1.6 |  |
| E | E | 0.5 | 0.6 | 0.6 | 0.6 | 0.6 | 0.7 | | 0.3 | 0.6 | 0.7 |  |
| % dominant taxon | % dom | 28.3 | 37.7 | 56.9 | 33.4 | 35.4 | 48.4 | | 41.6 | 47.3 | 92 |  |
| % Hirudinea | %Hir | 0 | 0.2 | 1 | 0.1 | 0.3 | 1.2 | | 0 | 0 | 0.5 |  |
| % Oligochaeta | %Oli | 1.1 | 9.3 | 16.7 | 2.5 | 6 | 13.8 | | 0.1 | 0.5 | 18.7 |  |
| % Crustacea | %Cru | 24.8 | 36.1 | 63.2 | 25.5 | 44.9 | 58.4 | | 30.1 | 47.7 | 97.7 |  |
| **% Amphipoda** | **%Amp** | **3** | **10.2** | **28** | **0.4** | **14.5** | **26** | | **0** | **0** | **3.9** |  |
| % EOT | %EOT | 0.8 | 2 | 8 | 0 | 1.3 | 2.8 | | 0 | 0 | 0.3 |  |
| % Ephemeroptera | %E | 0 | 0 | 0 | 0 | 0 | 0.1 | | 0 | 0 | 0 |  |
| % Ephemeroptera+Trichoptera | %ET | 0.4 | 0.6 | 2.9 | 0 | 0.3 | 1.9 | | 0 | 0 | 0.3 |  |
| % Dytiscidae | %Dyt | 1.6 | 3.8 | 7.9 | 0.8 | 2.2 | 3.6 | | 0.1 | 0.2 | 7.5 |  |
| % Diptera | %Dip | 8.4 | 13.4 | 18.9 | 6.9 | 15.1 | 28.8 | | 1.6 | 8.3 | 21.9 |  |
| % Chironomidae | %Chi | 7.5 | 12.6 | 17.7 | 6.3 | 15.1 | 28.7 | | 1.4 | 8.3 | 10.9 |  |
| % Orthocladiinae in Chironomidae | %Ort | 48.2 | 68.5 | 84.8 | 14.2 | 31.4 | 77.2 | | 21.4 | 60.9 | 90 |  |
| % Gastropoda | %Gas | 0 | 0 | 1.3 | 0 | 0 | 0 | | 0 | 0 | 0 |  |
| **% Predators** | **%P** | **3.7** | **19.1** | **22.8** | **5.3** | **10.2** | **14.2** | | **0.2** | **0.8** | **8.8** |  |
| % Scrapers | %Sc | 0.4 | 1 | 2.7 | 0 | 0.3 | 1.1 | | 0 | 0 | 0 |  |
| % Filterers | %F | 2.8 | 13 | 30.3 | 5.5 | 15.7 | 45.2 | | 11.3 | 40.3 | 91.5 |  |
| % Collector–gatherers | %CG | 35.4 | 53.1 | 75.1 | 33.1 | 55.1 | 65.4 | | 8 | 43.2 | 71.9 |  |
| % Shredders | %Sh | 0.4 | 0.7 | 2.6 | 0 | 0 | 0.3 | | 0 | 0 | 0 |  |
| % Piercers–herbivores | %PH | 0.2 | 1.3 | 2.9 | 0.1 | 3.4 | 12.1 | | 0 | 0 | 0.2 |  |
| % B Predators | %BP | 29.7 | 34.8 | 39.2 | 17.1 | 25.6 | 44.5 | | 2.5 | 9.2 | 54.3 |  |
| % B Scrapers | %BSc | 0.2 | 0.7 | 3.3 | 0 | 0.5 | 2.5 | | 0 | 0 | 0 |  |
| % B Filterers | %BF | 2.4 | 3.4 | 23.3 | 0.4 | 1.4 | 6.6 | | 0.3 | 1.4 | 70.2 |  |
| % B Collector–gatherers | %BCG | 21.1 | 27.8 | 45.1 | 25.3 | 40.7 | 66.5 | | 19.3 | 24.4 | 37.5 |  |
| % B Shredders | %BSh | 2 | 2.8 | 10 | 0 | 0 | 1.5 | | 0 | 0 | 0 |  |
| % B Piercers–herbivores | %BPH | 0.2 | 2.1 | 4.9 | 0.1 | 2.5 | 17.8 | | 0 | 0.1 | 5.2 |  |
